# Transition from pediatric to adult medical care – A survey in young persons with inflammatory bowel disease

**DOI:** 10.1371/journal.pone.0177757

**Published:** 2017-05-18

**Authors:** Antje Timmer, Jenny Peplies, Max Westphal, Birgit Kaltz, Antje Ballauff, Martin Claßen, Martin W. Laass, Sibylle Koletzko

**Affiliations:** 1 Division of Epidemiology and Biometry, Department of Health Services Research, Medical Faculty, Carl von Ossietzky University, Oldenburg, Germany; 2 Department of Clinical Epidemiology, Leibniz Institute for Prevention Research and Epidemiology – BIPS GmbH, Bremen, Germany; 3 Institute for Public Health & Nursing Research IPP, Faculty of Human and Health Sciences, Bremen, Germany; 4 German Crohn’s Disease and Ulcerative Colitis association (registered association), Berlin, Germany; 5 Center for Children’s and Adolescent Medicine, Krefeld, Germany; 6 Childrens Hospital, Klinikum “Links der Weser“, Bremen, Germany; 7 Children’s Hospital, Medical Faculty Carl Gustav Carus, Technical University, Dresden, Germany; 8 Division of Pediatric Gastroenterology and Hepatology, Dr. von Hauner Children’s Hospital, Ludwig Maximilian University, Munich, Germany; Yale University School of Medicine, UNITED STATES

## Abstract

**Background:**

Transition to adult health services is a vulnerable phase in young persons with chronic disease. We describe how young persons with inflammatory bowel disease in Germany and Austria experience care during the transitional age, focusing on differences by type of provider (pediatric vs. adult specialist, no specialist).

**Methods:**

This was a follow up survey in patients previously registered with a pediatric IBD registry. Patients aged 15 to 25 received a postal questionnaire, including a measure of health care experience and satisfaction. Descriptive analyses were stratified by age. Sub-analyses in the 18–20 year age group compared health care experience by type of provider. Determinants of early or late transfer were examined using multinomial logistic regression.

**Results:**

619 patients responded to the survey; 605 questionnaires were available for analysis. Usual age of completing transition was 18. Earlier transfer was more common with low parental SES (OR 1.8, 95% CI 0.7 to 4.6), and less common with advanced schooling (OR 0.5, 95% CI 0.2 to 1.2). Structured transition was uncommon. 48% of the respondents had not received any preceding transition advice. Overall satisfaction with IBD care was high, especially with respect to interpersonal aspects, but less so in aspects of continuity of care.

**Conclusions:**

Despite high overall patient satisfaction, relevant deficiencies in transitional care were documented. Some of these were associated with lower parental social status. Differences in health care satisfaction by type of provider (adult vs. pediatric) were small.

## Introduction

The care of children with chronic health problems poses very complex challenges in which the whole family is involved. As the child grows up, there is an increasing demand on autonomy, and the young adult will eventually have to take over responsibility for his or her health [1]. This shift is also reflected in the transition from pediatric to adult medical care. In this context, transition has been defined as “the purposeful, planned movement of adolescents and young adults with chronic physical and medical conditions from child-centered to adult-oriented healthcare systems” [2–4]. Transition should be considered a process rather than a point in time, in analogy to the psychosocial maturation process underlying the patient’s growing self-dependence. Readiness to transfer is a complex challenge involving, beside patients, parents and providers, a number of systemic and context issues [5–8]. Recommendations on how to prepare for transition address age groups from as young as 11 to 13 years [9]. However, failed transition, as reflected in poor health outcomes, insufficient knowledge and failure to attend a specialist following discharge from pediatric care has been documented as an almost universal problem across different health care systems and conditions [3, 7, 10, 11].

This also applies to the inflammatory bowel diseases (IBD), i.e. Crohn’s disease, ulcerative colitis and colitis unclassified [12–17]. The very unpredictable course and a wide variety of potential complications of IBD including manifestations of an embarrassing nature deeply impact on quality of life and life planning. Affective problems and delayed psychosocial development may be a consequence [18–20]. Various barriers to successful transition have been identified, mostly relating to insufficient self-efficacy in the young patients [5, 8, 14, 21–26].

Differences in the approach to patients by type of provider have also been considered problematic. For example, pediatricians are described to be more family centered and to take more time, as compared to adult service providers who may expect more autonomy on the part of the patient and typically have less office time available for individual patients [17, 27, 28]. This may lead to substantial anxiety in the young person (and parents) preceding transfer to adult care, relating to perceptions of lower quality care from adult providers [8, 12, 29, 30]. However, there has been little objective evidence so far to support substantial differences between pediatric and adult gastroenterologists [13, 31].

The current survey was performed to collect information on the situation of care in young persons with IBD in Germany. Specifically, we wished to describe at which age patients leave pediatric care, where they are subsequently treated and whether they are satisfied with the care they receive. Secondary analyses also presented in this paper focus on perceived differences between adult and pediatric care in the subgroup of 18 to 20 year olds.

## Material and methods

### Design and setting

The study was performed as a cross-sectional survey, following up patients previously registered in the German language pediatric inflammatory bowel disease registry, CEDATA-GPGE [32, 33]. This registry includes patients with physician confirmed IBD who were 0 to 17 at the time of first documentation and were seen at least once by one of the participating pediatricians in Germany and Austria.

### Patient recruitment and data collection

Patients were eligible for inclusion if aged 15 to 24 at the start of the recruitment period. Questionnaires were sent out to parents who had previously consented to be contacted. A single reminder to non-respondents was mailed after two weeks. Patients received a multi-modular pre-tested questionnaire which enquired about sociodemographic, clinical and health care related information.

Age at completing transition was defined as the age at which the person left pediatric care or started attending joint clinics. The German health system requires patients to have been transferred to adult services by the age of 18; exceptions apply. For this study, transfer up to age 17 was considered early completion of transition, transfer at 19 or later was considered late. The physician currently responsible for IBD related decisions (IBD care provider) was characterized as pediatric gastroenterologist (PG), adult gastroenterologist (GE), or “other” (e.g. non-GI specialist, general physician (GP) or general pediatrician).

Measures used to prepare for transfer were asked for based on a checklist which took into account recommendations from the literature and expert advice [15, 28, 34, 35]. An English version (ad hoc translation) of this module is provided as Supporting Information (S1 File). General satisfaction with medical IBD care was measured by a single global question on a 4 point scale. In addition, patient priorities and experience were evaluated based on a previously validated 32 item disease-specific patient satisfaction questionnaire [36]. In this instrument, single items are individually rated for both importance and the degree to which they are perceived as fulfilled, each on a four point scale (“not important” to “very important”, and “not met” to “fully met”). An English version of this instrument is available with our previous publication describing the validation of the questionnaire [36].

#### Sociodemographic variables and comorbidity

Current age was categorized as 15 to 17, 18 to 20, and 21 to 25 at the time of the survey. Parental socioeconomic status (SES) was calculated based on level of education, current occupation and household income of the parents [37, 38]. Schooling level was categorized as basic (up to 10 years of schooling, or left school without qualification), intermediate (10 years of schooling), advanced (qualifying for university entry), or unknown/other. In addition, type of living environment (rural, small town, urban; with parents, alone/with partner/with friends), job status (attending school/university, job training, and working) and type of insurance (statutory, private) were assessed. As relevant comorbidity, evidence of depressive or anxiety disorders was evaluated using the German version of the Hospital Anxiety and Depression Scale (HADS-D, cut-off = 11) [39, 40].

#### Disease specific variables

Type of disease included Crohn’s disease (CD), ulcerative colitis (UC) and IBD not specified (INS) based on the questionnaire information. INS comprised IBD with unclear or inconsistent information, as well as unclassified or indeterminate colitis. Age at onset was classified as onset in childhood (age 0 to 9), pre-adolescence (age 10–13) and adolescence (age ≥ 14). This categorization served as a proxy for disease duration, with the aim to untie the close correlation of this variable with current age. For disease activity, the survey modifications of the Crohn’s Disease Activity Index and the Colitis Activity Index (S-CDAI, S-CAI) were calculated [41]. Remission was defined as S-CDAI ≤ 150 for CD, and S-CAI ≤ 4 for UC and INS. Disease course during the preceding year (no relapse, one relapse, several relapses/persistent symptoms) was used to indicate severity of disease.

### Statistical analysis

**Fig 1:**
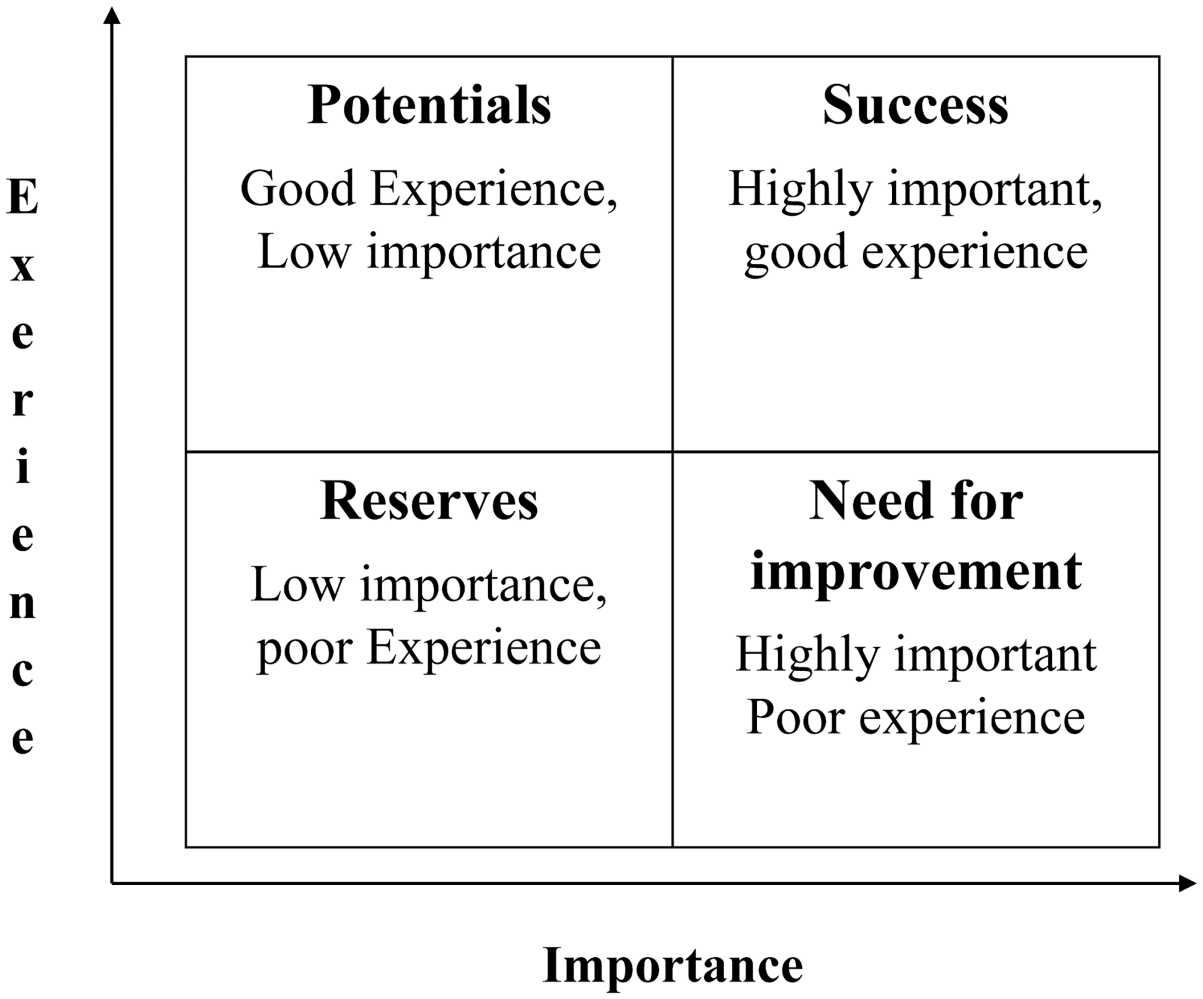
Correlation grid.

The main analyses were descriptive, showing absolute numbers and percentages for categorical or categorized variables, stratified by age group. Numbers for categories may not add up to totals where information was missing. Details of patient priorities and experience were graphically examined (Fig 1). Items were considered relevant if rated important or very important (“importance”) by at least 50% of the participants. Any item not perceived as met or fully met (“experience”) by at least 90% was considered as critical (in need of improvement), in particular if also considered relevant.

Determinants of early or late transfer were examined in the subset of patients > 18 years of age only, and were determined using multinomial logistic regression where the nominal variable specified the age at completing transition as either transfer at the age of 18 (reference), early (< 18) or late (> 18) completion. Owing to the trans-sectional design, selection of potentially influential variables into the model was restrictive to avoid inclusion of factors which may also be a consequence of quality of care or time of transition. Specifically we included sex, schooling, parental SES, age of onset and region within Germany. Variables with small numbers in relevant categories were either not considered (migration status), or cases in this category were excluded (region of residence: Austria). Since all independent variables in this model were categorical, exponential coefficient estimates may be interpreted as conditional odds ratios

$$\text{exp}\left(\beta_{ij}\right)=\;\frac{odds\text{(}Y=i|X=j\;and\;\left(Y=i\;or\;Y=r_{Y}\right))}{odds\text{(}Y=i|X=r_{X}\;and\;\left(Y=i\;or\;Y=r_{Y}\right))},$$

where *r*_*Y*_ is the reference category of the response variable Y and *r*_*X*_ is the reference category of the considered independent variable X. A formal prerequisite for this model is the Independence of Irrelevant Alternatives assumption implying that the (conditional) OR does not depend on the presence or absence of other categories of the dependent variable.

Additional sub-analyses were performed in the 18 to 20 year age group to examine differences in the perception of the quality of care by IBD care provider. All comparisons were exploratory. P-values relate to chi squared testing (two sided, alpha level 5%).

Analyses were performed using SPSS vs. 22, or SAS 9.4.

### Ethical considerations

Ethical approval for the postal survey was granted (Bremen University ethics committee, date of approval June 1, 2011). Written informed consent was secured after detailed written information by all participants, and their parents or guardians if aged below 18 years.

## Results

Overall, 1387 questionnaires were sent out, of which 619 were returned. The response was 48.2% following correction for invalid parental addresses. Responder analysis showed an effect of age on the response proportion (15 to 17: 53.5%, 18 to 20: 45.5%, 21 to 25: 64.6%), but not sex. Fourteen questionnaires were excluded due to incomplete information on disease, age or sex, resulting in 605 cases left for analysis. Of these, 212 were under-aged at the time of the survey (15 to 17), 255 were in the 18 to 20 year old group, and 138 were 21 years or older.

### Baseline description and differences by age

Basic information on the full sample is shown in Table 1. As expected, the proportion of persons living at home and going to school decreased with increasing age. Unemployment and inability to work were uncommon. 74.4% of all responders were in remission at the time of the survey and more than half had not had a relapse during the preceding 12 months. This was similar across all age groups.

**Table 1:** Patient characteristics by current age (n, column percent).

		15 to 17 years	18 to 20 years	21 to 25 years	All
**Sex**	**Male**	105 (49.5%)	140 (54.9%)	61 (44.2%)	306 (50.6%)
**Living situation**	**With parents**	205 (97.6%)	202 (80.8%)	54 (39.7%)	461 (77.3%)
**Job situation**^a^	**Still at school**	148 (70.1%)	69 (27.1%)	3 (2.2%)	220 (36.4%)
	**University**	1 (0.5%)	55 (21.8%)	52 (38.0%)	108 (18.2%)
	**Job training**	45 (21.8%)	83 (32.9%)	31 (22.6%)	159 (26.7%)
	**Working**	3 (1.5%)	17 (6.7%)	39 (28.5%)	59 (9.9%)
**Environment**^b^	**Rural**	95 (46.1%)	97 (39.8%)	28 (20.9%)	220 (37.7%)
	**Urban**	67 (32.5%)	75 (30.7%)	83 (61.9%)	225 (38.5%)
**Parental SES**	**Low**	31 (15.0%)	34 (13.8%)	23 (17.0%)	88 (14.9%)
	**Middle**	92 (44.4%)	121 (49.0%)	55 (40.7%)	268 (45.5%)
	**High**	84 (40.6%)	92 (37.2%)	57 (42.2%)	233 (39.6%)
**Type of schooling**	**Basic**	46 (21.7%)	33 (12.9%)	17 (12.3%)	96 (15.9%)
	**Intermediate**	68 (32.1%)	89 (34.9%)	52 (37.7%)	209 (34.5%)
	**Advanced**	98 (46.1%)	133 (52.2%)	69 (50.0%)	300 (49.6%)
**Health insurance**	**Privat**	36 (18.1%)	32 (13.0%)	21 (15.6%)	89 (15.3%)
**Comorbidity**	**Anxiety**	9 (4.4%)	16 (6.5%)	12 (9.4%)	37 (6.4%)
	**Depression**	3 (1.4%)	5 (2.0%)	8 (6.0%)	16 (2.7%)
**Type of IBD**	**Crohn’s Disease**	136 (64.1%)	179 (70.2%)	85 (61.6%)	400 (66.1%)
	**Ulcerative Colitis**	65 (30.7%)	65 (25.5%)	44 (31.9%)	174 (28.8%)
	**Not specified**	11 (5.2%)	11 (4.3%)	9 (6.5%)	31 (5.1%)
**Disease activity**	**Active**	40 (20.2%)	71 (29.1%)	36 (27.1%)	147 (25.6%)
	**Remission**	158 (79.8%)	173 (70.9%)	97 (72.9%)	428 (74.4%)
**Disease course**	**No relapse**	113 (54.6%)	139 (55.8%)	76 (55.5%)	328 (55.3%)
**(in preceding year)**	**1 relapse**	49 (23.7%)	59 (23.7%)	28 (20.4%)	136 (22.9%)
	**> 1 relapse**	45 (21.7%)	51 (20.4%)	33 (24.1%)	129 (21.7%)
**Age at diagnosis**	**0 to 9**	62 (30.7%)	53 (21.5%)	25 (19.7%)	141 (24.3%)
	**10 to 13**	108 (53.5%)	107 (43.3%)	57 (43.2%)	272 (46.8%)
	**14 +**	32 (15.8%)	87 (35.2%)	49 (37.1%)	168 (28.9%)
**Disease duration**	**< 2 years**	7 (4.5%)	2 (0.9%)	0 (0.0%)	9 (1.9%)
	**2 to 5 years**	87 (55.8%)	95 (44.8%)	7 (6.8%)	189 (40.1%)
	**> 5 years**	62 (39.7%)	115 (54.2%)	96 (93.2%)	273 (58.0%)
**Total**		212	255	138	605

^a^ Not shown: apprenticeship, internships, voluntary work, unemployed, unable to work

^b^ Not shown: small town

### Transition information

#### Age at completing transition, current provider of IBD care

Of 283 persons who were 19 or older, 48 (17%) had moved on to adult care by the age of 17 (early transfer), 128 (45.2%) left at age 18 (reference), and 87 (30.7%) started attending adult care at age 19 or older, or had not yet been through transition (late transfer) (missing: n = 20).

35 persons did not currently have a physician for IBD related consultations. This was most common in the oldest age group (13 persons, 9.7%; youngest age group: 1.4%, middle age group: 7.6%). The proportion of patients not under age appropriate IBD specialist care decreased with age: While 82% of the 15 to 17 year olds were seen by a PG, only 64% of those aged 21 and older were in GE care.

#### Determinants of early or late transfer

Persons with high parental SES or those attending/ having attended schools qualifying for university entry were most likely to complete transition late (Table 2). Late completion was particularly common in the South, and relatively rare in the East of Germany. However, there was a strong individual center variation within these regions. Proportions for late completion varied from 0 to 65% between all centers contributing 15 or more patients to this sub-analysis.

**Table 2:** Potential determinants of age at transfer (n, row percent; only cases aged > 18).

		Early (up to 17)	Reference (18)	Late (19 and later)	All
**Sex**	**Male**	20 (15.0%)	63 (47.4%)	50 (37.6%)	133
	**Female**	28 (21.5%)	65 (50.0%)	37 (28.5%)	130
**School / Exam**	**Basic /other**	4 (16.0%)	17 (68.0%)	4 (16.0%)	25
	**Intermediate**	25 (26.9%)	46 (49.5%)	22 (23.7%)	93
	**Advanced**	19 (13.1%)	65 (44.8%)	61 (42.1%)	145
**Age at Diagnosis**	**0 to 9**	12 (21.8%)	28 (50.9%)	15 (27.3%)	55
	**10 to 13**	19 (17.8%)	51 (47.7%)	37 (34.6%)	107
	**14 +**	16 (17.6%)	45 (49.5%)	30 (33.0%)	91
**Region (collapsed)**	**North-West**	11 (23.4%)	22 (46.8%)	14 (29.8%)	47
	**Middle-West**	11 (21.2%)	24 (46.2%)	17 (32.7%)	52
	**South-West**	9 (15.3%)	23 (39.0%)	27 (45.8%)	59
	**East**	17 (17.5%)	57 (58.8%)	23 (23.7%)	97
	**Austria**	0	2 (25.0%)	6 (75.0%)	8
**Parental SES**	**Low**	11 (30.6%)	20 (55.6%)	5 (13.9%)	36
	**Middle**	19 (17.4%)	64 (58.7%)	26 (23.9%)	109
	**High**	14 (12.6%)	42 (37.8%)	55 (49.5%)	111
**Type of IBD**	**Crohn’s Disease**	32 (18.7%)	83 (48.5%)	56 (32.7%)	171
	**Ulcerative Colitis**	13 (17.6%)	38 (51.4%)	23 (31.1%)	74
	**Not specifided**	3 (16.7%)	7 (38.9%)	8 (44.4%)	18
**Total**		48 (18.3%)	128 (48.7%)	87 (33.1%)	263

**Fig 2:**
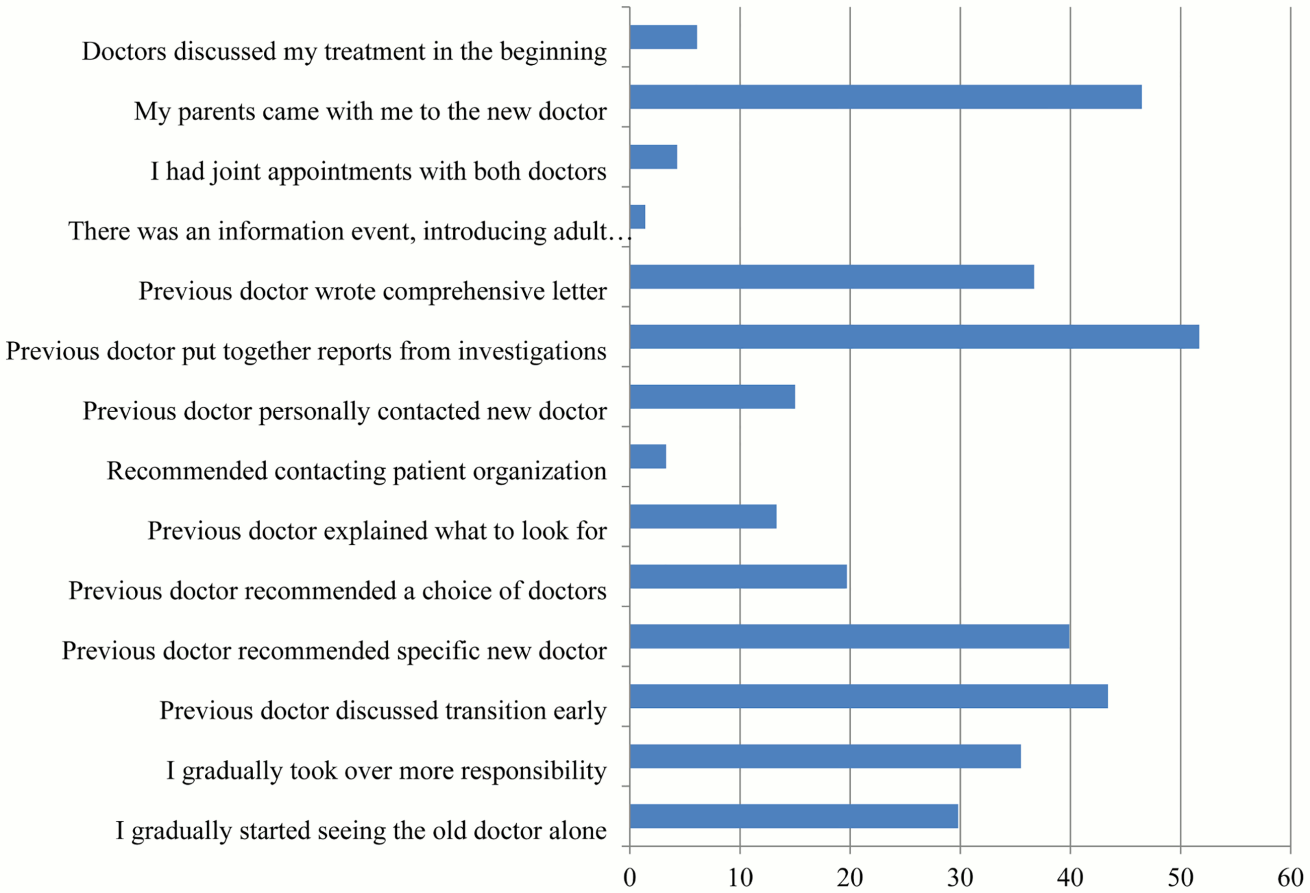
Determinants of early and late transfer (multinomial regression) (OR with 95% CI). Reference categories: male sex, intermediate schooling, age of onset 14+, middle region, middle SES, ulcerative colitis + not specified.

Schooling: Basic: Haupt/Volksschule, Hauptschulabschluss; Other: not given, Gesamtschule, special schools, other; intermediate: Realschule, Fachhochschulreife; advanced: A levels, Abitur, Matura, Gymnasium;

The results of the multinomial logistic regression are shown in Fig 2. All coefficients had large confidence intervals. Most obviously, transfer at 19 or later was common in persons with high parental SES (OR 3.2, 95% CI 1.6 to 6.4) and those living in the South of Germany (OR 1.7, 95% CI 0.7 to 4.2). Early transfer occurred most commonly in those with low parental SES (OR 1.8, 95% CI 0.7 to 4.6), and was particularly uncommon in persons undergoing advanced schooling (OR 0.5, 95% CI 0.2 to 1.2). Undergoing basic schooling only, and living in the East of Germany showed the highest propensity for being in the reference category, i.e. completing transition at age 18. There were no differences by age of onset (i.e. disease duration), sex and type of disease.

#### Measures taken to facilitate transition

346 persons had already left pediatric care, and 314 of these gave details on how the transition process had been initiated. In 179 cases (52%), the PG, or IBD doctor, had prepared the youngster for transition, in 18 cases (5%) the GP or general pediatrician. 23% reported they had organized it without help of a physician, 8% said the change just happened by chance, and 5 persons had not been in need of a physician recently and during the transitional stage.

Of 289 patients already transfered and giving a reason for change, 233 (81%) reported that transfer occurred because they had turned 18 years of age. 32 (11%) had changed for practical reasons, e.g. moving to another town, and 24 (8.3%) because they were not satisfied with the previous physician.

**Fig 3:**
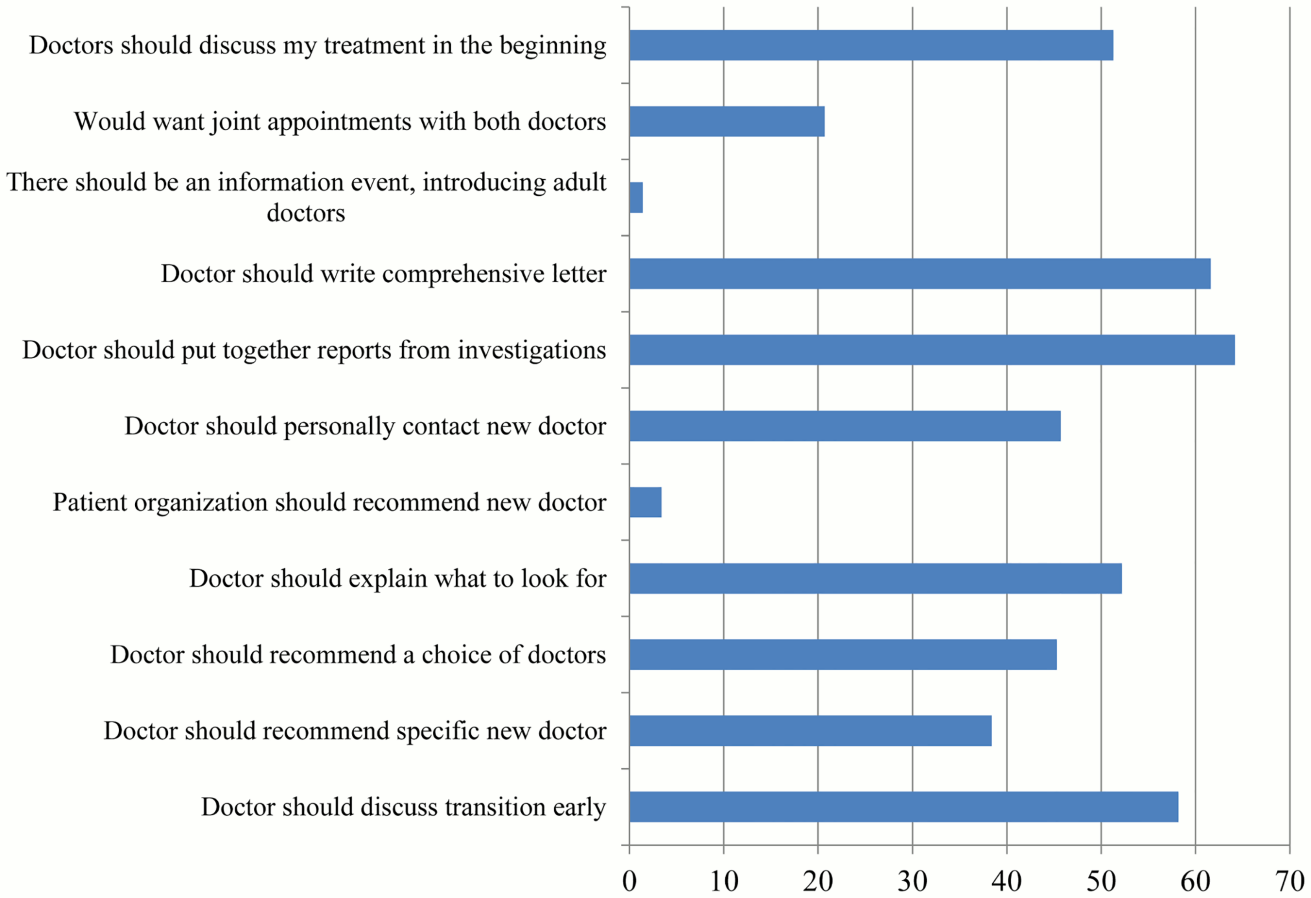
Measures taken by IBD doctor to prepare for transition (transferred patients only).

Measures taken to prepare for transfer, as reported by patients, are shown in Fig 3. None of the measures were reported by more than 52% of all patients. The most common preparative measures were the collection of previous reports by the pediatric caregiver, and the parents coming along to the new doctor. Joint appointments had been attended by 15 patients (4%). The majority of patients reported they were very satisfied (72; 21%) or satisfied (170; 49%) with the transition process (not satisfied: 12%, very unsatisfied: 6%).

### Current satisfaction with care

The satisfaction with the current IBD care was high: 539 of 583 who gave this information reported to be very satisfied or satisfied (92.5%) on global questioning. The proportion of persons not or not at all satisfied increased from 2.9% (6 persons) in the youngest age group to 12.8% (17 persons) in the oldest age group (middle age group: 8.6% (21 persons), or from 2.2% (n = 5) in those not yet transfered to 11.5% (n = 38) following transfer.

**Fig 4:**
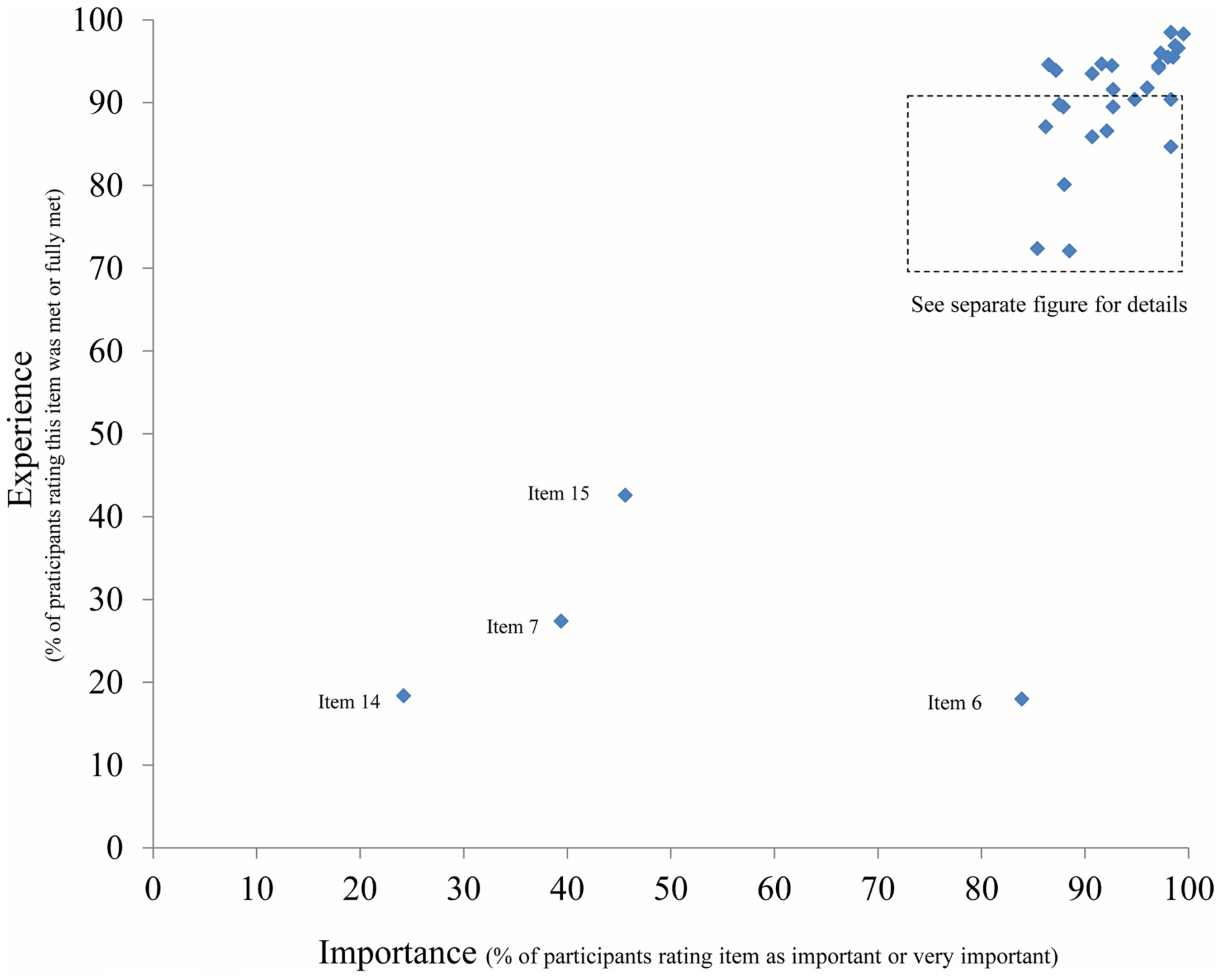
Patient satisfaction: Single item performance, overview (n = 583).

Single item analysis showed the majority of items in the upper right quadrant of the importance—experience grid (high importance, good experience) (Fig 4). Specifically, all items relating to interpersonal relationship, communication and patient autonomy, as well as cleanliness and availability of toilets were rated as both highly important and mostly met (> 90%). An acceptable taste of the cleansing solution for colonoscopy was considered very important by the majority of patients but very rarely met (item 6, Fig 4). Interdisciplinary care (psychologist, item 14 or other specialties, item 15) and meeting patients of similar age (item 7) were experienced by less than 50% of the respondents, but were mostly not rated important.

**Fig 5:**
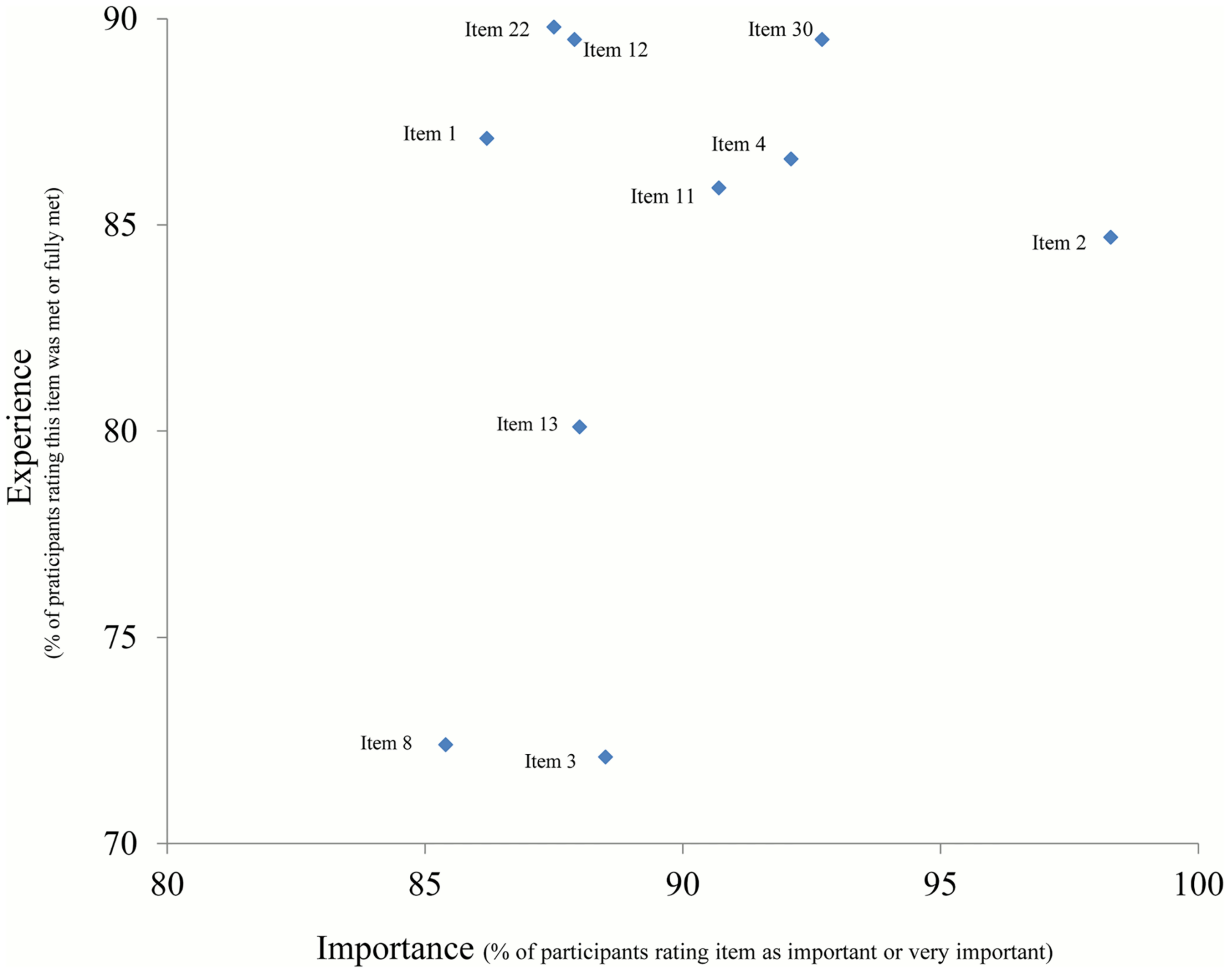
Single item performance, details: Items in need of improvement (n = 583).

There were 10 items which were considered important by more than 50% and perceived as met or fully met by between 70% and 90% of persons (Fig 5). These items are thus considered as in need of improvement and will be examined in more detail below for the middle age group, by type of provider. They mostly concerned aspects of inter-sectoral cooperation and practice organization.

### Single item experience: Differences by type of provider

245 persons were available for analysis in the subgroup of 18 to 20 year olds (136 male, 109 female). Of these 72 were seen by PG, 110 by adult GE and 63 by a non-specialist physician.

#### Comparison of patient groups

Baseline characteristics by type of provider are shown in Table 3. Most differences were small. Patients still at the pediatric specialist were relatively young within the age group, and most often of high parental SES. In contrast, patients seen by adult specialists had had more recent relapses, and were more frequently not in remission. This group also had the highest proportion of students, lived in urban areas more often, was more often privately insured, and tended to have had longer disease duration.

**Table 3:** Baseline characteristics by type of provider, 18 to 20 year olds only (n, column %).

		Paed. GE	Adult GE	Other	All
**Sex**	**Male**	41 (56.9%)	62 (56.4%)	33 (52.4%)	136 (55.5%)
**Age**	**18 years**	42 (58.3%)	33 (30.0%)	28 (44.4%)	103 (42.0%)
	**19 years**	20 (27.8%)	46 (41.8%)	21 (33.3%)	87 (35.5%)
	**20 years**	10 (13.9%)	31 (28.2%)	14 (22.2%)	55 (22.4%)
**Living Situation**	**With parents**	62 (87.3%)	82 (75.2%)	50 (82.0%)	194 (80.5%)
**Job Situation**	**Still at school**	27 (37.5%)	24 (22.0%)	16 (25.8%)	67 (27.6%)
	**University**	15 (20.8%)	31 (28.4%)	7 (11.3%)	53 (21.8%)
	**Job training**	20 (27.8%)	28 (25.7%)	31 (50.0%)	79 (32.5%)
	**Working**	4 (5.6%)	12 (1.8%)	1 (6.5%)	17 (2.5%)
**Environment**	**Rural**	30 (42.9%)	37 (34.6%)	24 (41.4%)	91 (38.7%)
	**Urban**	21 (30.0%)	38 (35.5%)	14 (24.1%)	73 (31.1%)
**SES**	**Low**	10 (14.3%)	10 (9.5%)	14 (22.6%)	34 (14.3%)
	**Middle**	21 (30.0%)	55 (52.4%)	37 (59.7%)	113 (47.7%)
	**High**	39 (55.7%)	40 (38.1%)	11 (17.7%)	90 (38.0%)
**Health Insurance**	**Privat**	9 (12.7%)	18 (17.0%)	4 (6.6%)	31 (13.0%)
**Comorbidity**	**Anxiety**	3 (4.3%)	5 (4.8%)	5 (9.5%)	14 (5.9%)
**Type of Disease**	**CD**	57 (79.2%)	77 (70.2%)	40 (63.5%)	174 (71.0%)
	**UC**	13 (18.1%)	29 (26.4%)	20 (31.7%)	62 (25.3%)
	**IC**	2 (2.9%)	4 (3.6%)	3 (4.8%)	9 (3.7%)
**Disease Activity**	**In Remission**	62 (87.3%)	81 (77.9%)	48 (81.4%)	191 (81.6%)
	**Active**	9 (12.7%)	23 (22.1%)	11 (18.6%)	43 (18.4%)
**Disease Course**	**No relapse**	40 (58.0%)	57 (52.3%)	38 (62.3%)	135 (56.5%)
	**1 relapse**	15 (21.7%)	29 (26.6%)	12 (19.7%)	56 (23.4%)
	**> 1 relapse**	14 (20.2%)	23 (21.1%)	11 (16.4%)	48 (20.0%)
**Age at Diagnosis**	**0 to 9**	13 (18.6%)	27 (25.5%)	11 (18.0%)	51 (21.5%)
	**10 to 13**	30 (42.9%)	47 (44.3%)	24 (39.3%)	101 (42.6%)
	**14 +**	27 (38.6%)	32 (30.2%)	26 (42.6%)	85 (35.9%)
**Disease Duration**	**< 5 years**	23 (36.5%)	25 (27.5%)	20 (40.0%)	68 (33.3%)
	**> 5 years**	40 (63.5%)	66 (72.5%)	30 (60.0%)	136 (66.7%)
**Total**		72	110	63	245

Those not in specialist care were most often in job training, e.g. apprenticeship. They had significantly more often a low parental SES, were least often privately insured, lived least often in an urban environment and showed the highest prevalence of anxiety, albeit on a low level insufficient for conclusive comparative analysis.

#### Experience of health care provision

**Fig 6:**
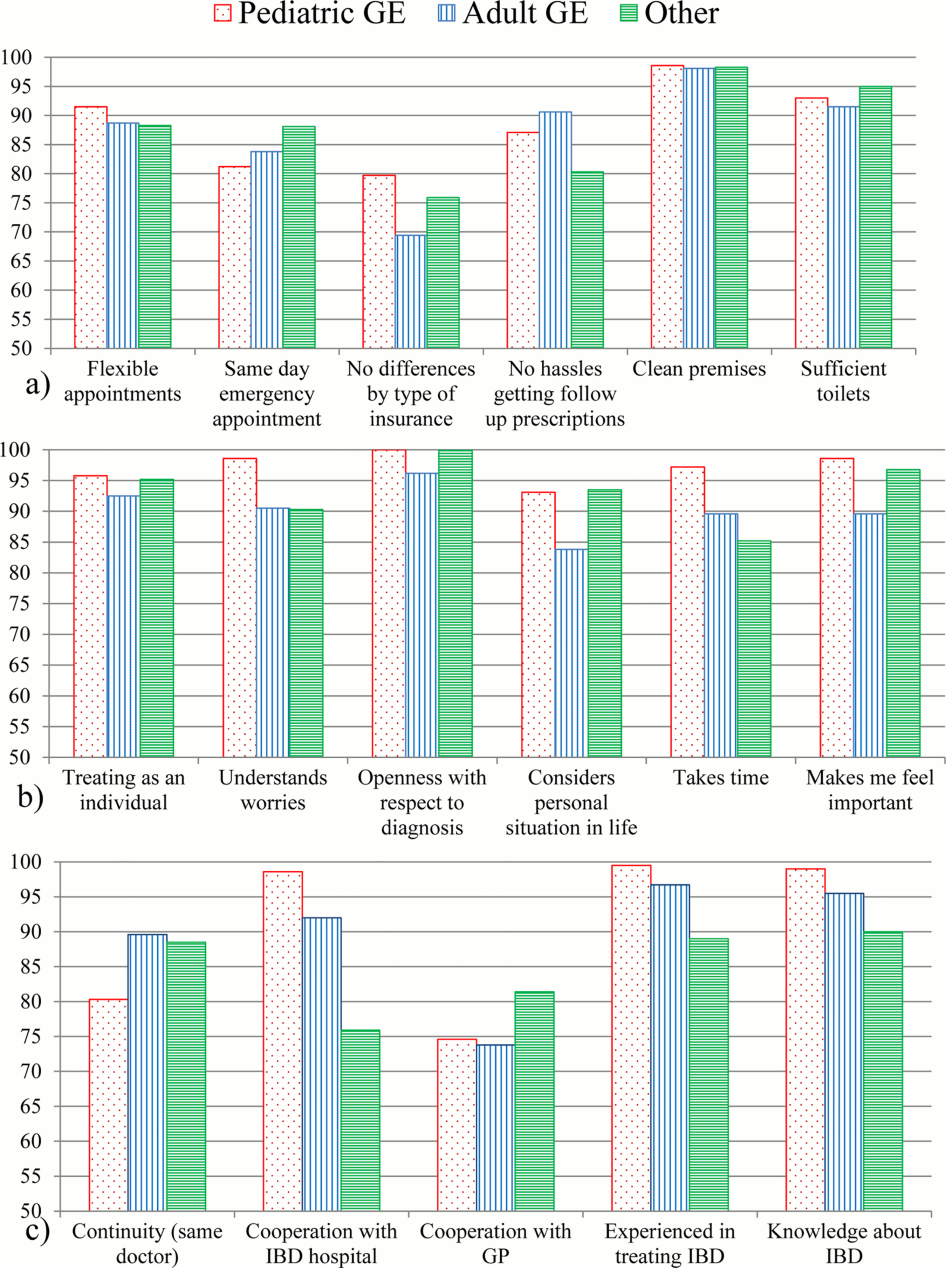
Experience of care by type of provider, middle age group only. a) Practice organization, logistics. b) Communication, patient-physician relationship issues. c) Professional cooperation and competence.

Single item experience by type of provider is shown in Fig 6. Across all types of providers, transparency of communication and issues of hygiene were generally considered excellent (> 90% in all groups), while equality in care with respect to insurance status, cooperation with the GP and continuity of care were most critical. Differences by provider were minor, and mostly not statistically significant. As compared to the other groups, PG were more often perceived as devoting sufficient time to the individual patient, taking worries and concerns seriously, taking patients’ schedules (e.g. school, exams) into account when making appointments (flexibility), making the young patient feel important and showing good cooperation with IBD clinics. This group performed worst with respect to continuity of care and same day emergency appointments. GE were slightly less often perceived to be knowledgeable in IBD as compared to pediatric specialists. They showed the lowest values as compared to both other groups with respect to not giving priority to privately insured patients, taking the personal life situation of the patient into account and making the patient feel important.

The non-specialists showed better cooperation with the GP and the best availability of same day emergency appointments. In this group, there were marked deficiencies with respect to cooperation with inpatient care providers, IBD competence and the provision of follow up prescriptions.

## Discussion

We were able to present detailed information on the current situation of care based on a large survey of more than 600 IBD patients in the transitional age group from across Germany and Austria. At first sight, the overall situation seems reassuring. The majority of patients were currently well with respect to disease activity and were actively engaged in studies or job. Few suffered from emotional problems. Most patients were satisfied with present care and with how transfer had taken place. Overall, all types of providers scored high on communication skills and other physician-patient-relationship-issues.

However, the high patient satisfaction stands in contrast to the low percentage of patients having experienced specific preparation for transition, as is now recommended by clinical guidelines [9, 15, 35, 42]. Almost a quarter of those already in adult care had organized transition without any help of a physician. Less than half of the patients reported there had been a comprehensive hand over letter to the new doctor; little more than 50% reported the previous doctor had put together reports from investigations. Personal contact between child provider and adult provider was uncommon, as were joint clinics. Only one in three young patients reported that they had started seeing the pediatrician alone preceding transfer. Interpretation has to take into account that this information is patient based. Physician initiated activities may not be comprehensively acknowledged, in particular as patient expectations seemed to be low. However, the low prevalence of perceived activities to prepare for transfer is striking.

Even more problematic, a quarter of patients in the middle age group, and a third beyond the age of 21 were not under specialist GI care for IBD related decisions. This was particularly common in those with low parental SES. The prominent role of parental SES as the single significant determinant of age of transfer may be in part due to differences in life planning, e.g. leaving school early (known to correlate with low parental SES in Germany). Serious concerns as to fair health care arise adding to this previously known evidence of social inequality. We have recently completed a study in pediatric IBD including health economic analyses to examine this issue further in younger patients [43].

There is some controversy in whether the timing of transfer should be guided chronologically, e.g. by certain age limits, or based on milestones, such as graduation, an approach which seems to be more appreciated by pediatricians [6, 14, 21, 44–46]. In Germany, the educational system is organized on state level (rather than federal), and the usual age of leaving school varies depending on region of residence. The differences in age of transfer we see with respect to region and type of schooling reflect these differences: the most common age of leaving school is 19 in most states, and 18 in some, mostly Eastern states. There is an obvious mismatch here between the milestone triggering change to adult care (graduation) at age 19, and the legally required age of transfer by age 18, which only affects persons attending advanced secondary schooling, in particular in the Western states.

Within the 18 to 20 year old group, patients seeing adult gastroenterologists had more severe disease as compared to those remaining in pediatric care. It seems plausible that patients, in particular those still at school, delay seeing a physician if in remission; experiencing relapse will then trigger ad hoc transfer to adult care. The pattern of differences between GE and PG, e.g. PG scoring worse on continuity of care but better in competence in IBD, is suggestive of a higher degree of specialization and academic environment in the PG as compared to the GE. PG also had substantial problems to provide same day emergency appointments. We checked our data following this finding and found, in addition, that pediatric specialists were farther away from the patients’ residence, were less often accessible by foot or bike as compared to adult gastroenterologists and had longer waiting times for appointments. All this, again, suggests, that patients (or their parents) take quite some time and effort to see an IBD specialized PG, even if this is logistically more difficult, while for the GE, often, the one conveniently found in the neighborhood makes do. We suggest that more guidance in choosing the appropriate adult IBD physician is warranted, which may well mean longer travelling times to specialized IBD units [47]. Information on physicians with specific interest and expertise in IBD is easily available e.g. from physician networks or the patient organization (www.kompetenznetz-ced.de; www.dccv.de). In our survey, patients had rarely received this information from their PG preceding transfer.

There are a number of limitations to our study. Foremost, this was a follow up study of patients from a registry run by pediatric gastroenterologists with specific interest in IBD. The registry is estimated to include about a third of all pediatric patients affected by IBD in Germany, less in Austria. Selection of patients with above average pediatric specialist care is likely, and some deterioration in satisfaction with care upon change to adult providers may represent regression to the mean. Selection effects are further aggravated by the low response rate. Due to logistic problems within the registry concurring with the start of our survey, we were not able to fully employ response-enhancing measures, such as 2nd reminders and phone calls to non-responders. Also, we had to approach patients via parents, and may have oversampled those remaining in close contact with home (and the pediatrician). Also, we cannot exclude that in some instances parents interfered with youth answering the questionnaire, or even answering instead of their children. In fact the study team was surprised by the number of parents (rather than patients themselves) calling our helpline or sending additional letters sharing experiences or enquiring about the study.

Response proportions in survey research have been described to be higher in those with either particularly low or particularly high satisfaction with care [48]. We feel that the chosen access via the pediatric registry may have led to a selection of patients particularly attached to the pediatric provider. Also, all information was patient based, and social desirability bias may be a possible additional factor favoring satisfaction with pediatric care.

Lastly, the cross sectional design hampered causal inference. Differences by provider are difficult to assess as the timing of change and the choice of provider is so intertwined with age and disease activity. Thus, all analyses have to be considered explorative. However, we feel that the detailed descriptive presentation has helped to illuminate how care in the transitional age group is currently perceived by those affected.

In conclusion, while the overall situation of young patients with IBD appeared to be satisfactory, relevant deficiencies in transitional care were identified. Ad hoc relapse-triggered choice of the adult care providers seemed common. In addition, a substantial number of young adults with IBD were not under specialist care at all.

Given the frequent lack of adherence to recommended transition preparation strategies in our sample, postponing legal age thresholds to synchronize provider change with late graduation from secondary school will not solve problems of insufficient self-efficacy as commonly described in IBD patients. It is appreciated that there is no financial compensation for transitional care, apart from some pilot regions and conditions [49], and that the evidence on the effectiveness of transition programs is still scarce [4].

Since this survey was performed, recommendations have been published to improve transitional care. Well tested structural transition procedures are currently being introduced in Germany, including compensation schemes from statutory health insurance [50, 51]. However, these will only work if the need to prepare for transfer is appreciated by all concerned. It seems important to foster positive attitudes towards autonomy and adult care in patients, parents and providers to improve participation in these programs. We suggest that the measures we used to assess the quality of care will be helpful to evaluate improvement in subsequent evaluations.

## Supporting information

S1 File Transition Survey Part H (Ad hoc English Translation of German Original). (PDF)

## Abbreviations

CD: Crohn’s diseases
CEDATA-GPGE: German language clinical registry for pediatric IBD patients
GE: Gastroenterologist
GI: gastrointestinal
GP: General Practitioner
HADS-D: German version of the Hospital Anxiety and Depression Scale
IBD: Inflammatory bowel diseases
INS: IBD not specified (unclear IBD, colitis unclassified, unclear colitis)
IQR: Interquartile range
IRB: Institutional Review Board
PG: Pediatric gastroenterologist
S-CAI: Survey-based Colitis Activity Index
S-CDAI: Survey-based Crohn’s Disease Activity Index
SES: Socioeconomic status
UC: Ulcerative colitis
