# Supplementary material for: Transition from pediatric to adult medical care – A survey in young persons with inflammatory bowel disease
Source: PLoS One. 2017 May 18;12(5):e0177757. doi: 10.1371/journal.pone.0177757 (PMC5436761; doi:10.1371/journal.pone.0177757)
Supplement: S1 File — (PDF) [file pone.0177757.s001.pdf]

H Doctor for Children or Doctor for Adults?

This part of the questionnaire deals with your experience with your IBD doctor during child- and adult care. We are particularly interested in whether you have changed from a pediatrician to an adult doctor or are still planning to do so. Also we wish to know how the change from child care to adult care took place, or is currently taking place.

1. Which statement applies to your situation? *(Please tick only one answer.)*
Questions relate to your IBD doctor. This is the doctor who is in charge of treating you IBD.

- ☐1 I have always been with a bowel specialist (gastroenterologist) for adults
- ☐2 I am still with a bowel specialist for children (pediatric gastroenterologist).
- ☐3 I changed from a pediatric specialist to a specialist for adults.
- ☐4 I am currently under care by both a children's and an adult's specialist.
- ☐5 I am not under specialist IBD care.
- ☐6 Other:____

2. If you have changed from a doctor for children to a doctor for adults:

a) How old were you when the change took place?

I__I__I Years of age ☐1 Did not change

b) What was the <u>most important reason</u> for you to change?
(Please tick only one answert.)

- ☐1 I could not remain with the children's doctor because I was of age
- ☐2 Practical reasons (e.g. moving place, access problems, change of personal circumstances)
- ☐3 I was not satisfied at the children's doctor for medical reasons / competence
- ☐4 I was not satisfied at the children's doctor for other reasons

c) Thinking back, would you have rather changed earlier or later?

- ☐1 I would have preferred to change later
- ☐2 I would have preferred to change earlier (at age I__I__I)
- ☐3 The timing was just right

3. Who took (or is taking) main charge of preparing you to change to adult care?

☐1 The general physician (GP) or general pediatrician

☐2 The pediatric gastroenterologist / previous IBD doctor

☐3 Nobody, I (and/or my parents) had to look after things ourselves

☐4 The change just happened by chance.

☐5 I had not been to a doctor for several months preceding the change.

☐6 other:____

4. What did (does) your <u>previous doctor</u> do to prepare for your change?
(Please check all applicable answers.)

☐1 I started seeing the previous doctor more and more on my own (without my parents).

☐1 I had to take more and more responsibility for managing my disease myself.

☐1 My doctor discussed with me early on, that and why a change was necessary.

☐1 He recommended a specific specialist for adults to me.

☐1 He recommended several specialists for adults I might choose from.

☐1 He explained to me what to check for if choosing a new doctor.

☐1 He recommended getting support from the patient organization.

☐1 He personally contacted the new doctor (e.g. by phone).

☐1 He compiled my patient notes (e.g. reports from investigations) for the new doctor.

☐1 He wrote a detailed transfer-letter about the previous course of my IBD.

☐1 There was an information event where several doctors for adults were introduced

☐1 There are joint clinics of the previous and the new doctor.

☐1 In the beginning, a friend of mine accompanied me to the new doctor.

☐1 In the beginning, my father or/and my mother accompanied me to the new doctor.

☐1 The previous and the new doctor consult with each other prior to and following the change.

☐1 None of this applies.

5. How satisfied were you overall with how the change had been prepared for? (or is being prepared)?

☐1 very satisfied ☐2 satisfied

☐3 not satisfied ☐4 very unsatisfied

6. What preparations would you want to have done prior to changing to adult care? If you have already changed: which preparations did you find / would you have found particularly helpful?

(Please check all applicable answers)

☐1 Early information why and when transfer has to take place.

☐1 Recommendation of a specific doctor.

☐1 Recommendation of several doctors to choose from

☐1 Information on what I have to check for if choosing a new doctor

☐1 Choosing a new doctor based on information provided by the patient organization

☐1 An information event where several doctors for adults are introduced.

☐1 My previous doctor personally contacting the new doctor, e.g. by phone

☐1 Compilation of relevant patient notes for the new doctor (e.g. results from investigations).

☐1 A detailed transfer letter describing the previous course of my disease.

☐1 Joint clinics by the previous and the new doctor.

☐1 Previous and new doctor discussing my treatment around the time of transfer

☐1 Other ideas: ______________________________

____

Please feel free to let us know your experiences during your transition phase in your own words. We have left space for this at the end of the questionnare: What did you find particularly helpful? What was particularly problematic? Do you have any suggestions on how the situation during the transitional phase could be improved?
